# Out with the old, in with the new: Assessing change in screen time when measurement changes over time

**DOI:** 10.1016/j.pmedr.2017.12.008

**Published:** 2017-12-24

**Authors:** Katie E. Gunnell, Jennifer Brunet, Mathieu Bélanger

**Affiliations:** a Department of Psychology, Carleton University, Canada; b School of Human Kinetics, University of Ottawa, Canada; c Epidemiology and Public Health, University of Ottawa, Canada; d Healthy Active Living and Obesity Research Group, Children's Hospital of Eastern Ontario Research Institute, Canada; e Institut de Recherche de l'Hôpital Montfort (IRHM), Hôpital Montfort, Canada; f Cancer Therapeutic Program, Ottawa Hospital Research Institute (OHRI), Canada; g Department of Family Medicine, Université de Sherbrooke, Canada; h Centre de Formation Médicale du Nouveau-Brunswick, Canada; i Vitalité Health Network, Canada

**Keywords:** Technology, Measurement, Screen time, Computers, Longitudinal, Adolescents, Shifting indicators, Invariance

## Abstract

We examined if screen time can be assessed over time when the measurement protocol has changed to reflect advances in technology. Beginning in 2011, 929 youth (9–12 years at time one) living in in New Brunswick (Canada) self-reported the amount of time spent watching television (cycles 1–13), using computers (cycles 1–13), and playing video games (cycles 3–13). Using longitudinal invariance to test a shifting indicators model of screen time, we found that the relationships between the latent variable reflecting overall screen time and the indicators used to assess screen time were invariant across cycles (weak invariance). We also found that 31 out of 37 indicator intercepts were invariant, meaning that most indicators were answered similarly (i.e., on the same metric) across cycles (partial strong invariance), and that 28 out of 37 indicator residuals were invariant indicating that similar sources of error were present over time (partial strict invariance). Overall, across all survey cycles, 76% of indicators were fully invariant. Whereas issues were noted when new examples of screen-based technology (e.g., iPads) were added, having established partial invariance, we suggest it is still possible to assess change in screen time despite having changing indicators over time. Although it is not possible to draw definitive conclusions concerning other self-report measures of screen time, our findings may assist other researchers considering modifying self-report measures in longitudinal studies to reflect technological advancements and increase the precision of their results.

## Highlights

• A screen time measurement protocol changed over time to reflect new technology.
• Despite a changing measurement protocol, most indicators/items were invariant over time.
• Change in ST could be assessed over time with caution at later time points.
• The shifting indicators model can be used to examine measures that change.

## 1 Introduction

Screen time (ST), or the time youth spend using screen-based devices such as watching television (TV) or using a computer, is ubiquitous. As media and technology advances are made, youth shift their ST behaviours and adopt new ones (Steeves, 2014). These shifts in ST behaviours pose a unique problem when researchers are attempting to track or assess change in overall ST over time. In this paper, we examined if ST can be compared over time when the indicators used to assess ST and the examples provided to participants, either verbally during questionnaire completion or in writing within the questionnaire, were adapted across assessments to reflect technological advancements.

### 1.1 Measurement of screen time

Measures of ST are widely incorporated into psychological, public health, epidemiological, and educational research because higher ST is related to negative physical and mental health outcomes (Carson et al., 2016, Tremblay et al., 2011). ST is often assessed using self-report questionnaires, whereby participants are asked to report the number of hours they engaged in specific types of screen-based behaviours over a specified period of time (Gunnell et al., 2016, Kremer et al., 2014, Lacy et al., 2011). A key factor that may influence the ability of existing self-report questionnaires to capture ST is the rapid pace at which screen-based technology is evolving. For example, researchers may be hesitant to make modifications to their assessments to capture new screen-based technologies because of the widespread notion that one must use identical measures to assess change over time (Lloyd et al., 2009). Rather, it is common practice to use the same ST measures repeatedly and examine changes in participants' responses. Nevertheless, failure to modify indicators or examples to account for new screen-based devices or screen-based behaviours may lead to an underestimation (or overestimation) of ST in some participants. For example, imagine a 12-year-old who reports watching 4 h of TV and playing 1 h of video games daily (total ST = 5 h). Now, as a 15-year-old and given advancements in portable technology, they spend 1 h watching TV on a stationary television set and playing video games, and 4 h engaging in various tablet-based behaviours (e.g., watching videos, surfing the web, playing interactive games; total ST = 5 h). If, however, the ST measure used in the first assessment was not modified to include (a) either a new indicator directly assessing time spent on a tablet or (b) tablets as example screen-behaviours in an existing indicator in the second assessment, the 15-year old might not think to report their time spent on the tablet. As a consequence, it might appear as though their ST had decreased over time although it did not change (i.e., if there was no place for the youth to report tablet use, the measure is not accurately capturing overall ST). This example highlights the notion that using the same measures with static and possibly outdated examples over time may be inappropriate when it comes to measuring ST.

With the recognition that ST measures must be updated alongside technological advancements, new indicators and/or examples within each indicator have been added to existing surveys. For example, the Canadian Health Measures Survey asked participants to estimate their usage of smartphone and tablets in a recent survey whereas previous surveys did not include these indicators of ST (Government of Canada, 2016). Similarly, the Monitoring Activities of Teenagers to Comprehend their Habits (MATCH; Bélanger et al., 2013) study added an indicator asking participants to report time spent playing video games and provided additional examples (i.e., tablets and smartphones) for one of the indicators in the questionnaire used for later data collection cycles. To-date, researchers have yet to examine if change in ST over time can be calculated despite changes in the measurement within longitudinal studies. Using data from the MATCH study, we examined if ST can be examined over time if modifications were made to how ST was assessed to account for shifts in ST technology. Modifications were twofold: (1) adding a new indicator in the questionnaire and (2) adding new examples of ST to the questionnaire rather than verbally cuing participants to include those types of ST. In other words, in early survey cycles, children and youth were verbally instructed to include tablets and smartphones when answering the ST indicators. The instruction was provided verbally because these devices were uncommon at the time (in 2011). Over time, tablet and smartphone use became more prevalent and as a consequence, the measurement protocol changed to provide these screen-based devices as written examples (rather than verbal) in the assessment of screen time.

## 2 Methods

### 2.1 Participants and procedures

The MATCH study began in 2011/2012. Initially, 802 youth who were 9–12 years old were recruited from 17 urban and rural schools in New Brunswick; however, the number of participants increased to 936 (*N* = 929 with ST data) because new participants of the same age cohort were allowed to join the study after study inception. The procedures have been presented in more detail elsewhere (Bélanger et al., 2013). Briefly, 19 out of 21 schools contacted agreed to participate. Two schools were subsequently excluded due to low return of consent forms leaving 17 schools. Participants from these 17 schools completed either French or English questionnaires during class time in the presence of a trained research assistant. At cycle 1, 51% of the students agreed to participate in the study. At cycle 1, questionnaires took approximately 45–60 min to complete with follow-up questionnaires lasting about 20–30 min. Questionnaires were completed approximately every 4 months during the school year coinciding with Fall, Winter, and Spring.

At the time of analyses, data from cycles 1–13 were analyzed; this included all data collected throughout the school year up until Fall 2015. Ethics approval was granted by Comité d'Éthique de la Recherche du Centre Hospitalier de l'Université de Sherbrooke, and all participants provided written informed assent and their parents provided written informed consent.

### 2.2 Measure

From cycles 1–13, ST was assessed using two indicators (Utter et al., 2003) reflecting time spent: (a) watching TV and videos, and (b) using a computer (not for homework). From cycles 3–13, ST was assessed using these two indicators (a and b) as well as time spent (c) playing video games such as XBOX, Nintendo, and Playstation. An indicator of time spent playing video games was added in cycle 3 and onwards after realizing video game playing was a salient screen behaviour that was inadvertently omitted from cycles 1 and 2. In cycles 1–8, participants were verbally instructed to consider time spent using all types of screen-based devices such as iPod, iPhone, iPad, or tablets. In cycle 9 and onwards, these examples were added in writing to questions (a) and (b) described above. Participants reported the number of hours spent doing each activity separately for weekend days (Saturday–Sunday) and weekdays (Monday–Friday) using the following response options: 1 (0 h), 2 (1/2 hour), 3 (1 h), 4 (2 h), 5 (3 h), 6 (4 h), and 7 (5 h or more). Consistent with previous research (Gunnell et al., 2016, Utter et al., 2003), we created a weighted score for each ST behaviour (i.e., weighted watching TV and videos = [5 ∗ weekday] + [2 ∗ weekend]; weighted using the computer = [5 ∗ weekday] + [2 ∗ weekend]; weighted playing video games = [5 ∗ weekday] + [2 ∗ weekend]). Researchers have demonstrated score reliability of ST indicators with youth through test-retest correlations ranging from 0.69 to 0.80 (Utter et al., 2003).

### 2.3 Data analysis

Initially, data were screened for univariate outliers (*z* scores > 3.3) and the calculation of descriptive statistics were carried out in SPSS (Version 23) to describe the sample and detect any deviation from normality. Next, using Mplus 7.3 with robust maximum likelihood estimation (MLR), the shifting indicators model (Hancock and Buehl, 2008) was used to determine if a latent variable reflecting overall ST is operating the same over time, despite changes in individual indicators. The shifting indicator model was used for several reasons. First, it uses a confirmatory factor analytic technique that is generally well known to researchers and relatively easy to implement using various statistical software programs (Bandalos and Raczynski, 2015). Second, the shifting indicator model allows researchers to scale all indicators, whether they are the same or not across time, in a standardized way to facilitate comparisons across time (Bandalos and Raczynski, 2015).

Within the shifting indicators model, longitudinal invariance constraints were added to common indicators over time (Hancock and Buehl, 2008, Lloyd et al., 2009, Widaman et al., 2010). Extending classic applications of longitudinal invariance, the shifting indicator model relies on the assumption that there are sets of common indicators at adjacent time points (Hancock and Buehl, 2008), but all indicators do not have to appear in the measure at all time points (see Bandalos and Raczynski, 2015, Hancock and Buehl, 2008). First, to confirm the appropriateness of a ST latent variable at each cycle, we specified correlated latent variables of ST at each time point to load onto their respective calculated scores from watching TV and videos (cycles 1–13), using the computer (cycle 1–13) and playing video games (cycles 3–13) within a confirmatory factor analysis. In all models, errors of identical indicators were permitted to covary across time points. Next, we tested for measurement invariance in common indicators across time (e.g., watching TV and videos across cycles 1–13; using the computer across cycles 1–13; playing video games across cycles 3–13). Levels of invariance tested were: (a) item factor loadings (i.e., *weak* invariance), (b) item intercepts (i.e., *strong* invariance), and (c) item residuals (i.e., *strict* invariance; Mplus syntax is available in Appendix 1; Hoffman, 2016). Weak invariance is tested to verify that all common indicators are comparably salient for overall ST across cycles (Bandalos and Raczynski, 2015). Strong invariance is tested to determine if the same amount of overall ST, on average, elicits the same responses by participants to the response scale (Bandalos and Raczynski, 2015). If the intercepts are not similar across cycles and a specific mean level of overall ST is associated with different mean levels of the outcome at different time points, it becomes impossible to examine mean differences across time due to differential scaling of ST. Strict invariance is estimated to determine if the residuals (i.e., errors) from each indicator are equivalent across cycles and can therefore be compared across cycles (Gregorich, 2006).

In testing levels of invariance, we compared the more constrained to less constrained models (see evaluation criteria below). If there were no significant decreases in fit, we interpreted the results as evidence of full invariance, indicating that subsequent statistical techniques to examine change over time could be employed despite the change in indicators used to assess ST behaviours (Bandalos and Raczynski, 2015, Hancock and Buehl, 2008).

#### 2.3.1 Assessing model fit and comparing nested models

Comparative fit index (CFI) values close to or above 0.90 and a Root Mean Square Error of Approximation (RMSEA) close to or below 0.06 were used to determine good model fit (Brown, 2006, Hu and Bentler, 1999). Parameter estimates were also examined for magnitude and out of range values (e.g., standardized values above 1). When comparing more constrained models against less constrained models to determine if invariance in the parameters was found, we considered a ΔCFI | < 0.01 | and ΔRMSEA | < 0.015 | as a non-significant decrement in fit (Chen, 2007, Cheung and Rensvold, 2002). Of note, finding a lack of full invariance is common, and in such cases partial invariance can be examined (Byrne et al., 1989, Gregorich, 2006, Marsh et al., 2010, Steenkamp and Baumgartner, 1998). Thus, if significant decrements in model fit were observed, suggesting full invariance could not be established, we examined partial invariance by freeing one constrained parameter at a time based on modification indices until the model met ΔCFI | < 0.01 | and ΔRMSEA | < 0.015 | (Byrne et al., 1989).

## 3 Results

Missing data, largely attributable to study design (i.e., recruitment remained open after cycle 1 so participants recruited at cycle 2 had missing data at cycle 1, etc.) ranged from 24.3% to 44.2% (*M* = 35%). No univariate outliers were identified (*z*-scores < 2.82). The current analyses included 929 youth (68.0% who completed questionnaires in French; 55.5% girls) who were 10.34 (SD = 0.646, *n* = 606) years old at study inception (Fall 2011), and 11.29 (SD = 0.66, *n* = 690), 12.21 (SD = 0.70, *n* = 657), 13.25 (SD = 0.71 *n* = 570), and 14.22 years old (SD = 0.72, *n* = 535) in Fall 2012, 2013, 2014, and 2015, respectively. As estimated through linking residential postal codes reported in 2014/2015 to area-level income obtained from the 2006 Canadian Census, the mean income of parents of the participants included in the current analyses was CAD$31,053 (SD = $8309, *n*_missing_ = 432). Means and standard deviations are presented in Table 1, whereas medians, skewness values (range = | 0.06–1.24 |), and kurtosis values (range = | 0.03–1.08 |) are presented in the online Appendix (see Table 2).

**Table 1:** Descriptive statistics across each survey cycle.

	TV/videos		Computer use		Video games	
	M	SD	M	SD	M	SD
Cycle 1	24.79	10.39	19.77	10.38	–	–
Cycle 2	26.18	10.14	20.22	10.77	–	–
Cycle 3	24.61	9.89	19.29	10.87	17.42	11.98
Cycle 4	25.31	9.80	19.76	11.06	17.17	12.02
Cycle 5	25.45	9.72	20.46	11.85	19.58	13.43
Cycle 6	25.27	10.36	20.51	12.61	18.68	13.82
Cycle 7	24.97	9.86	19.40	12.29	17.71	12.99
Cycle 8	25.60	10.53	20.43	12.73	18.90	13.87
Cycle 9	24.41	10.35	25.65	12.70	22.78	13.63
Cycle 10	25.42	10.73	27.08	12.35	22.75	13.92
Cycle 11	25.25	10.23	27.75	12.81	23.39	14.13
Cycle 12	24.61	10.86	27.95	12.59	22.76	14.34
Cycle 13	25.06	10.92	28.91	12.96	22.03	14.94

*Notes:* TV = television, M = mean, SD = standard deviation. Units of measurement for TV, computer use and video games are not hours per week. Scores range from 7 to 49. Cycle 1 was conducted in Fall 2011 and the MATCH study was carried out in New Brunswick (Canada).

**Table 2:** Results of invariance test for screen time.

	MLRχ^2^_(df)_	CFI	ΔCFI	RMSEA	RMSEA 90%CI	ΔRMSEA
No constraints	381.95⁎_(340)_	0.995	–	0.012	[0.00, 0.02]	–
Loadings constrained	419.63⁎_(362)_	0.994	0.001	0.013	[0.01, 0.02]	0.001
Intercepts constrained	731.19⁎_(384)_	0.963	0.031	0.031	[0.03, 0.04]	0.018
TV/Videos cycle 9 freed	681.34⁎_(383)_	0.968	0.026	0.029	[0.03, 0.03]	0.016
Computers cycle 13 freed	654.89⁎_(382)_	0.971	0.026	0.028	[0.02, 0.03]	0.015
Computers cycle 12 freed	633.56⁎_(381)_	0.973	0.021	0.027	[0.02, 0.03]	0.014
TV/videos cycle 11 freed	601.15⁎_(380)_	0.976	0.018	0.025	[0.02, 0.03]	0.012
TV/videos cycle 10 freed	539.55⁎_(379)_	0.983	0.011	0.021	[0.02, 0.03]	0.008
TV/videos cycle 12 freed	498.30⁎_(378)_	0.987	0.007	0.019	[0.01, 0.02]	0.006
Residuals constrained	640.04⁎_(406)_	0.975	0.012	0.025	[0.02, 0.03]	0.006
Computers cycle 8 freed	625.38⁎_(405)_	0.976	0.011	0.024	[0.02, 0.03]	0.005
TV/Videos cycle 13 freed	612.77⁎_(404)_	0.977	0.010	0.024	[0.02, 0.03]	0.005
Video games cycle 13 freed	602.61⁎_(403)_	0.978	0.009	0.023	[0.02, 0.03]	0.004

*Notes:* CFI = comparative fit index, RMSEA = root mean square error of approximation. Cycle 1 was conducted in Fall 2011 and the MATCH study was carried out in New Brunswick (Canada).

^⁎^ p < .05

Results of the CFA with no equality constraints across cycles (i.e., configural model) confirmed the appropriateness of the ST latent variables as the model fit the data well (see Table 2). Adding constraints to the factor loadings (i.e., regression coefficients between each indicator and overall ST) confirmed that each ST indicator was salient for measuring overall ST across cycles (i.e., weak invariance; ΔCFI = 0.001, ΔRMSEA = 0.001; see Table 2). Adding constraints to each indicator common intercepts decreased model fit significantly (see Table 2), meaning full strong invariance was not tenable. After freeing intercept constraints one at a time based on modification indices, the retained model had equality constraints on 31 out of the 37 possible intercepts, providing evidence for partial strong invariance. The intercepts for the indicators of watching TV and videos at cycles 9–12 and of computer use at cycles 12–13 were not invariant meaning that these indicators were not scaled identically across time. Nevertheless, after freeing these indicators intercepts, partial invariance was confirmed. Having found that 84% of the indicators had common scaling across cycles (i.e., intercepts were invariant), overall ST was considered to be largely measured on the same metric across cycles, indicating that participants responded similarly to the response scale across cycles. Next, constraining the common residuals of the indicators that had constrained common intercepts over time resulted in a significant decrement in fit (see Table 2). After freeing residual constraints one at a time based on modification indices, the final model had equality constraints on all common residuals except for the indicators of watching TV/videos at cycles 9–13, using the computer at cycles 8, 12 and 13, and playing video games at cycle 13 (see Table 2), meaning that for the error in these indicators differed across cycles. These results demonstrate partial strict invariance as 26 out of the 37 indicators had similar sources of error influencing them over time. In summary, most indicators (76%) were fully invariant even after a new indicator (i.e., playing video games) was added at cycle 3. Nonetheless, non-invariance in some intercepts and residuals appeared to coincide with the addition of written examples of iPod, iPhone, iPad, or tablets at cycle 9 and onward.

## 4 Discussion

Modifying measures or measurement protocols of ST is necessary given the ever-changing nature of digital media and technology. We demonstrated that across 13 data collection cycles spanning 4 years, most of the indicators (76%) used to assess ST had similar meaning, were answered on similar metrics, and had similar sources of error over time. Given that only a small percent of the indicators used were non-invariant, we have confidence that mean scores in overall ST can be examined over time. Nevertheless, our findings point to some differences in (a) how youth answered the response scales, and (b) the sources of error that influenced indicators at cycles when new written examples were included (i.e., iPod, iPhone, iPad, or tablet).

Given that full invariance could not be established, we tested partial invariance (Marsh et al., 2010, Vandenberg and Lance, 2000). Our results showed that only 24% (9 out of 27) of the indicators were non-invariant. There is no consensus on how many indicators can be non-invariant (Gregorich, 2006, Hancock and Buehl, 2008, Little, 2013, Marsh et al., 2010, Widaman et al., 2010) or how many are needed to allow for mean comparisons in ST scores across time. However, using the same approach as other researchers (Marsh et al., 2010) and following suggestions that partial invariance may be permissible when the majority of indicators are fully invariant (Little, 2013), our results of partial invariance indicate that we could still analyze data collected with a measure of ST that was modified at different times throughout a longitudinal study to assess change over time, albeit with a degree of caution, especially at later survey cycles.

The timing of the non-invariant indicators appeared commensurate with the written addition of examples cuing participants to think about iPod, iPhone, iPad, or tablets. Although these examples were provided verbally during early survey administration (cycles 1–8), it is possible that participants forgot the verbal instruction when it came time to complete the ST indicators. It would be useful to use qualitative methods such as think aloud procedures (cf. Zumbo and Hubley, 2017) to determine how and why participants were responding to indicators in the manner that they were. Alternatively, it might be useful to employ item response theory to determine under what circumstances indicators are demonstrating different functioning.

### 4.1 Practical implications and future directions

Researchers working within contexts involving digital media and technology will continue to be confronted by issues associated with longitudinal measurement. The approach used in the MATCH study to include indicators to assess use of emerging screen-based devices in writing after previously only included them verbally was consistent with ongoing data collection through the Canadian Health Measures Survey (Government of Canada, 2016). Although it is not possible to draw definitive conclusions concerning other self-report measures of indicators, our findings may assist other researchers considering modifying self-report measures in longitudinal studies to reflect technological advancements and increase the precision of results. In turn, the shifting indicators model (Bandalos and Raczynski, 2015, Hancock and Buehl, 2008) is one viable method they could use to examine the impact of measuring variables that may have changing indicators over time (e.g., new devices).

To make sure researchers capture the breadth of ST behaviours, there are at least three areas of research that warrant careful attention. First, new indicators may need to be added to confirm new devices, such as virtual reality gaming, are accounted for in measures of ST. Second, because youth engage in screen multi-tasking (e.g., watching TV while simultaneously using a smartphone) more research is necessary to ensure measures reflect trends in screen multi-tasking (Tremblay et al., 2011). Third, screens are multi-functional (e.g., a computer can be used to play video games and watch TV shows) and researchers will need to make sure their measures account for such cross-screen behaviours. In turn, adaptations to measures made based on these three pertinent issues around ST can be quantitatively examined using the shifting indicators model to confirm that overall ST is being assessed unambiguously over time.

### 4.2 Limitations

All data were collected via self-report measures which are susceptible to recall bias and social desirability responding. Further, given the indicators of ST used, we were unable to determine how participants responded when they used each type of screen for more than one purpose (e.g., played video games on computers). In future research, it may be beneficial to have direct measures of screen use (e.g., software applications that directly monitor screen usage). Additionally, although the purpose of our paper was to examine if we can assess change in overall ST over time despite changes in the measures and measurement protocol, we recommend that researchers further investigate ST measures to determine if they are operating similarly across different groups (e.g., different sexes, socioeconomic statuses). Also, the participants from the MATCH study were from one province; therefore, the results may not be generalizable to other youth living in other provinces in Canada or countries. Finally, although we used sophisticated data analytic procedures to handle missing data, missing data were present at all survey cycles and could have affected the results.

## 5 Conclusions

Given our findings that most of the indicators assessing ST across cycles in the MATCH study were invariant, we suggest that ST can be measured across time, despite changes in the indicators and measurement protocol. Nevertheless, we did find that the additional written examples of new technology (i.e., iPod, iPhone, iPad, or tablet) caused differences in how youth answered the questions and differences in the sources of error influencing indicators. Designing measures and adapting measures in longitudinal investigations to address the fluid nature of screen-based technology is a fruitful and necessary area of inquiry especially when linking ST to health outcomes.

## Conflict of interest

The authors have no conflicts of interest to declare.

## Appendix 1 Supplementary data

Model syntax for each level of invariance and median, skewness and kurtosis values. Image 1
