# Supplementary material for: Out with the old, in with the new: Assessing change in screen time when measurement changes over time
Source: Prev Med Rep. 2017 Dec 24;9:37–41. doi: 10.1016/j.pmedr.2017.12.008 (PMC5766751; doi:10.1016/j.pmedr.2017.12.008)
Supplement: Supplementary file 1 — Model syntax for each level of invariance and median, skewness and kurtosis values. [file mmc1.doc]

APPENDIX A

Model syntax for each level of invariance

**TITLE: CONFIGURAL INVARIANCE**

DATA: FILE IS SHIFTING_MPLUS_2.csv;

VARIABLE:

NAMES ARE ID TV1-TV13 COMP1-COMP13 VID3-VID13;

IDVARIABLE are ID;

USEVARIABLES ARE

TV1-TV13 COMP1-COMP13 VID3-VID13;

MISSING ARE ALL (999);

ANALYSIS:

Estimator =MLR;

MODEL:

!FACTOR LOADINGS ALL FREE RIGHT NOW

sed1 by comp1 tv1; !(L1-L2);

sed2 by comp2 tv2; !(L1-L2);

sed3 by comp3 tv3 vid3; !(L1-L3);

sed4 by comp4 tv4 vid4; !(L1-L3);

sed5 by comp5 tv5 vid5; !(L1-L3);

sed6 by comp6 tv6 vid6; !(L1-L3);

sed7 by comp7 tv7 vid7; !(L1-L3);

sed8 by comp8 tv8 vid8; !(L1-L3);

sed9 by comp9 tv9 vid9; !(L1-L3);

sed10 by comp10 tv10 vid10; !(L1-L3);

sed11 by comp11 tv11 vid11; !(L1-L3);

sed12 by comp12 tv12 vid12; !(L1-L3);

sed13 by comp13 tv13 vid13; !(L1-L3);

!MEANS OF THE LATENT VARIABLES ALL FREE RIGHT NOW

[sed1-sed13*];

!INTERCEPTS OF THE FIRST INDICATOR FOR EACH LATENT VARIABLE

!SET TO ZERO

[comp1-comp13@0];

!ALL OTHER INTERCEPTS ALL FREE RIGHT NOW

[tv1-tv13];!(I1);

[vid3-vid13]; !(I2);

!RESIDUALS ALL FREE RIGHT NOW

tv1-tv13; !(E1);

comp1-comp13; ! (E2);

vid3-vid13; !(E3);

!ITEM ERROR CORRELATIONS OVER TIME

tv1 with tv2-tv13;

tv2 with tv3-tv13;

tv3 with tv4-tv13;

tv4 with tv5-tv13;

tv5 with tv6-tv13;

tv6 with tv7-tv13;

tv7 with tv8-tv13;

tv8 with tv9-tv13;

tv9 with tv10-tv13;

tv10 with tv11-tv13;

tv11 with tv12-tv13;

tv12 with tv13;

vid3 with vid4-vid13;

vid4 with vid5-vid13;

vid5 with vid6-vid13;

vid6 with vid7-vid13;

vid7 with vid8-vid13;

vid8 with vid9-vid13;

vid9 with vid10-vid13;

vid10 with vid11-vid13;

vid11 with vid12 vid13;

vid12 with vid13;

comp1 with comp2-comp13;

comp2 with comp3-comp13;

comp3 with comp4-comp13;

comp4 with comp5-comp13;

comp5 with comp6-comp13;

comp6 with comp7-comp13;

comp7 with comp8-comp13;

comp8 with comp9-comp13;

comp9 with comp10-comp13;

comp10 with comp11-comp13;

comp11 with comp12-comp13;

comp12 with comp13;

OUTPUT: STDYX tech4 modindices;

**TITLE: WEAK INVARIANCE**

DATA: FILE IS SHIFTING_MPLUS_2.csv;

VARIABLE:

NAMES ARE ID TV1-TV13 COMP1-COMP13 VID3-VID13;

IDVARIABLE are ID;

USEVARIABLES ARE

TV1-TV13 COMP1-COMP13 VID3-VID13;

MISSING ARE ALL (999);

ANALYSIS:

Estimator =MLR;

MODEL:

!COMMON FACTOR LOADINGS CONSTRAINED ACROSS TIME POINTS

sed1 by comp1* tv1(L1-L2);

sed2 by comp2* tv2 (L1-L2);

sed3 by comp3* tv3 vid3(L1-L3);

sed4 by comp4* tv4 vid4(L1-L3);

sed5 by comp5* tv5 vid5(L1-L3);

sed6 by comp6* tv6 vid6(L1-L3);

sed7 by comp7* tv7 vid7(L1-L3);

sed8 by comp8* tv8 vid8(L1-L3);

sed9 by comp9* tv9 vid9(L1-L3);

sed10 by comp10* tv10 vid10(L1-L3);

sed11 by comp11* tv11 vid11(L1-L3);

sed12 by comp12* tv12 vid12(L1-L3);

sed13 by comp13* tv13 vid13(L1-L3);

!MEANS OF THE LATENT VARIABLES ALL FREE RIGHT NOW

[sed1-sed13*];

!FACTOR VARIANCES: FIRST FIXED TO 1 FOR IDENTIFICATION, ALL OTHER FREE

sed1@1;

sed2-sed13*;

!INTERCEPTS OF THE FIRST INDICATOR FOR EACH LATENT VARIABLE SET TO !ZERO

[comp1-comp13@0];

!ALL OTHER INTERCEPTS ALL FREE RIGHT NOW

[tv1-tv13];!(I1);

[vid3-vid13]; !(I2);

!RESIDUALS ALL FREE RIGHT NOW

tv1-tv13; !(E1);

comp1-comp13; ! (E2);

vid3-vid13; !(E3);

!ITEM ERROR CORRELATIONS OVER TIME

tv1 with tv2-tv13;

tv2 with tv3-tv13;

tv3 with tv4-tv13;

tv4 with tv5-tv13;

tv5 with tv6-tv13;

tv6 with tv7-tv13;

tv7 with tv8-tv13;

tv8 with tv9-tv13;

tv9 with tv10-tv13;

tv10 with tv11-tv13;

tv11 with tv12-tv13;

tv12 with tv13;

vid3 with vid4-vid13;

vid4 with vid5-vid13;

vid5 with vid6-vid13;

vid6 with vid7-vid13;

vid7 with vid8-vid13;

vid8 with vid9-vid13;

vid9 with vid10-vid13;

vid10 with vid11-vid13;

vid11 with vid12 vid13;

vid12 with vid13;

comp1 with comp2-comp13;

comp2 with comp3-comp13;

comp3 with comp4-comp13;

comp4 with comp5-comp13;

comp5 with comp6-comp13;

comp6 with comp7-comp13;

comp7 with comp8-comp13;

comp8 with comp9-comp13;

comp9 with comp10-comp13;

comp10 with comp11-comp13;

comp11 with comp12-comp13;

comp12 with comp13;

OUTPUT: STDYX tech4 modindices;

**TITLE: PARTIAL STRONG INVARIANCE**

DATA: FILE IS SHIFTING_MPLUS_2.csv;

VARIABLE:

NAMES ARE ID TV1-TV13 COMP1-COMP13 VID3-VID13;

IDVARIABLE are ID;

USEVARIABLES ARE

TV1-TV13 COMP1-COMP13 VID3-VID13;

MISSING ARE ALL (999);

ANALYSIS:

Estimator =MLR;

MODEL:

!COMMON FACTOR LOADINGS CONSTRAINED ACROSS TIME POINTS

sed1 by comp1* tv1(L1-L2);

sed2 by comp2* tv2 (L1-L2);

sed3 by comp3* tv3 vid3(L1-L3);

sed4 by comp4* tv4 vid4(L1-L3);

sed5 by comp5* tv5 vid5(L1-L3);

sed6 by comp6* tv6 vid6(L1-L3);

sed7 by comp7* tv7 vid7(L1-L3);

sed8 by comp8* tv8 vid8(L1-L3);

sed9 by comp9* tv9 vid9(L1-L3);

sed10 by comp10* tv10 vid10(L1-L3);

sed11 by comp11* tv11 vid11(L1-L3);

sed12 by comp12* tv12 vid12(L1-L3);

sed13 by comp13* tv13 vid13(L1-L3);

!FACTOR MEANS: FIRST FIXED TO ZERO FOR IDENTIFICATION, ALL OTHER !MEANS FREE

[sed1@0];

[sed2-sed13*];

!FACTOR VARIANCES: FIRST FIXED TO 1 FOR IDENTIFICATION, ALL OTHER FREE

sed1@1;

sed2-sed13*;

!INTERCEPTS ALL CONSTRAINED EXCEPT NON-INVARIANT INTERCEPTS

[comp1-comp11] (I1);

[tv1-tv8 tv13](I2);

[vid3-vid13] (I3);

[comp12-comp13*];

[tv9-tv12*];

!RESIDUALS ALL FREE RIGHT NOW

tv1-tv13; !(E1);

comp1-comp13; ! (E2);

vid3-vid13; !(E3);

!ITEM ERROR CORRELATIONS OVER TIME

tv1 with tv2-tv13;

tv2 with tv3-tv13;

tv3 with tv4-tv13;

tv4 with tv5-tv13;

tv5 with tv6-tv13;

tv6 with tv7-tv13;

tv7 with tv8-tv13;

tv8 with tv9-tv13;

tv9 with tv10-tv13;

tv10 with tv11-tv13;

tv11 with tv12-tv13;

tv12 with tv13;

vid3 with vid4-vid13;

vid4 with vid5-vid13;

vid5 with vid6-vid13;

vid6 with vid7-vid13;

vid7 with vid8-vid13;

vid8 with vid9-vid13;

vid9 with vid10-vid13;

vid10 with vid11-vid13;

vid11 with vid12 vid13;

vid12 with vid13;

comp1 with comp2-comp13;

comp2 with comp3-comp13;

comp3 with comp4-comp13;

comp4 with comp5-comp13;

comp5 with comp6-comp13;

comp6 with comp7-comp13;

comp7 with comp8-comp13;

comp8 with comp9-comp13;

comp9 with comp10-comp13;

comp10 with comp11-comp13;

comp11 with comp12-comp13;

comp12 with comp13;

OUTPUT: STDYX tech4 modindices;

**TITLE: PARTIAL STRICT INVARIANCE**

DATA: FILE IS SHIFTING_MPLUS_2.csv;

VARIABLE:

NAMES ARE ID TV1-TV13 COMP1-COMP13 VID3-VID13;

IDVARIABLE are ID;

USEVARIABLES ARE

TV1-TV13 COMP1-COMP13 VID3-VID13;

MISSING ARE ALL (999);

ANALYSIS:

Estimator =MLR;

MODEL:

!COMMON FACTOR LOADINGS CONSTRAINED ACROSS TIME POINTS

sed1 by comp1* tv1(L1-L2);

sed2 by comp2* tv2 (L1-L2);

sed3 by comp3* tv3 vid3(L1-L3);

sed4 by comp4* tv4 vid4(L1-L3);

sed5 by comp5* tv5 vid5(L1-L3);

sed6 by comp6* tv6 vid6(L1-L3);

sed7 by comp7* tv7 vid7(L1-L3);

sed8 by comp8* tv8 vid8(L1-L3);

sed9 by comp9* tv9 vid9(L1-L3);

sed10 by comp10* tv10 vid10(L1-L3);

sed11 by comp11* tv11 vid11(L1-L3);

sed12 by comp12* tv12 vid12(L1-L3);

sed13 by comp13* tv13 vid13(L1-L3);

!FACTOR MEANS: FIRST FIXED TO ZERO FOR IDENTIFICATION, ALL OTHER !MEANS FREE

[sed1@0];

[sed2-sed13*];

!FACTOR VARIANCES: FIRST FIXED TO 1 FOR IDENTIFICATION, ALL OTHER FREE

sed1@1;

sed2-sed13*;

!INTERCEPTS ALL CONSTRAINED EXCEPT NON-INVARIANT INTERCEPTS

[comp1-comp11] (I1);

[tv1-tv8 tv13](I2);

[vid3-vid13] (I3);

[comp12-comp13*];

[tv9-tv12*];

!RESIDUALS ALL CONSTRAINED EXCEPT NON-INVARIANT RESIDUALS

comp1-comp7 comp9-comp11 (E1);

tv1-tv8(E2);

vid3-vid12 (E3);

comp12-comp13*;

comp8*;

tv9-tv13*;

vid13*;

!ITEM ERROR CORRELATIONS OVER TIME

tv1 with tv2-tv13;

tv2 with tv3-tv13;

tv3 with tv4-tv13;

tv4 with tv5-tv13;

tv5 with tv6-tv13;

tv6 with tv7-tv13;

tv7 with tv8-tv13;

tv8 with tv9-tv13;

tv9 with tv10-tv13;

tv10 with tv11-tv13;

tv11 with tv12-tv13;

tv12 with tv13;

vid3 with vid4-vid13;

vid4 with vid5-vid13;

vid5 with vid6-vid13;

vid6 with vid7-vid13;

vid7 with vid8-vid13;

vid8 with vid9-vid13;

vid9 with vid10-vid13;

vid10 with vid11-vid13;

vid11 with vid12 vid13;

vid12 with vid13;

comp1 with comp2-comp13;

comp2 with comp3-comp13;

comp3 with comp4-comp13;

comp4 with comp5-comp13;

comp5 with comp6-comp13;

comp6 with comp7-comp13;

comp7 with comp8-comp13;

comp8 with comp9-comp13;

comp9 with comp10-comp13;

comp10 with comp11-comp13;

comp11 with comp12-comp13;

comp12 with comp13;

OUTPUT: STDYX tech4 modindices;

APPENDIX B

Table 2

*Medians, Skewness and Kurtosis Values A*cross each Survey Cycle

| Cycle | TV/Videos | | | Computer Use | | | Video Games | | |
| --- | --- | --- | --- | --- | --- | --- | --- | --- | --- |
|  | Median | Skew | Kurt | Median | Skew | Kurt | Median | Skew | Kurt |
| Cycle 1 | 23.0 | 0.64 | -0.21 | 16.0 | 1.03 | 0.64 | -- | -- | -- |
| Cycle 2 | 25.0 | 0.56 | -0.25 | 18.0 | 0.96 | 0.37 | -- | -- | -- |
| Cycle 3 | 23.0 | 0.55 | -0.09 | 16.0 | 0.96 | 0.36 | 14.0 | 1.24 | 0.68 |
| Cycle 4 | 23.0 | 0.51 | -0.29 | 16.0 | 0.92 | 0.16 | 14.0 | 1.17 | 0.42 |
| Cycle 5 | 23.0 | 0.52 | -0.17 | 18.0 | 0.85 | -0.04 | 14.0 | 0.88 | -0.44 |
| Cycle 6 | 23.0 | 0.57 | -0.21 | 18.0 | 0.80 | -0.34 | 14.0 | 1.06 | -0.15 |
| Cycle 7 | 23.0 | 0.57 | -0.11 | 16.0 | 0.96 | -0.03 | 14.0 | 1.12 | 0.08 |
| Cycle 8 | 23.0 | 0.51 | -0.18 | 17.0 | 0.90 | -0.17 | 14.0 | 0.97 | -0.34 |
| Cycle 9 | 23.0 | 0.49 | -0.16 | 23.0 | 0.37 | -0.90 | 21.0 | 0.55 | -0.90 |
| Cycle 10 | 24.0 | 0.41 | -0.37 | 25.5 | 0.30 | -0.94 | 21.0 | 0.49 | -0.99 |
| Cycle 11 | 23.0 | 0.52 | -0.07 | 27.0 | 0.18 | -1.00 | 22.0 | 0.41 | -1.08 |
| Cycle 12 | 23.0 | 0.50 | -0.22 | 28.0 | 0.19 | -0.97 | 21.0 | 0.53 | -0.99 |
| Cycle 13 | 24.5 | 0.34 | -0.36 | 28.0 | 0.06 | -1.08 | 19.0 | 0.58 | -1.06 |

*Note:* TV = television, skew = skewness values, kurt = kurtosis values. Units of measurement for TV, computer use and video games are not hours per week. Scores range from 7-49. Cycle 1 was conducted in Fall 2011 and the MATCH study was carried out in New Brunswick (Canada).
